# Adhesion of vessel wall to stentriever during combined technique for mechanical thrombectomy in acute ischemic stroke: A histomorphological study

**DOI:** 10.1177/15910199231216764

**Published:** 2023-12-06

**Authors:** Frank Donnerstag, Christopher Werlein, Friedrich Götz, Omar Abu Fares, Peter Raab, Enrico Calvino Iglesias, Heinrich Lanfermann, Mike P Wattjes, Danny Jonigk

**Affiliations:** 1 Institute for Diagnostic and Interventional Neuroradiology, 9177 Hannover Medical School, Hannover, Germany; 2 Institute for Pathology, 9177 Hannover Medical School, Hannover, Germany; 3 Biomedical Research in End-stage and Obstructive Lung Disease Hannover (BREATH) of the German Center for Lung Research (DZL), Hannover, Germany

**Keywords:** Stroke, thrombectomy, stentriever, clot, vessel wall, histology

## Abstract

**Purpose:**

Detection of vessel wall tissue in thrombus material in patients with ischemic stroke is judged as vascular injury. So far, several studies investigated components of the free clots after mechanical thrombectomy. The aim of this retrospective study was to investigate the involvement and role of the stentriever in vessel wall injury by analysis of the composition of adherent tissue to the stentriever during combined aspiration thrombectomy with stentriever.

**Methods:**

Stentriever with adherent tissue and free clots in aspiration samples from patients undergoing mechanical thrombectomy (aspiration plus stentriever) were separately assessed for the occurrence of parts of vascular tissue together with clinical and interventional data as well as clinical outcome data. Specimens were analyzed histomorphologically and immunohistochemically. Findings, focused on parts of vessel wall were reported together with clinical data.

**Results:**

Specimens from 21 identified patients were available. Parts of the vessel wall were detected in 7 out 21 (33%) samples. All specimens revealed fresh thrombus material without signs of organization or atheromatous tissue. In 90% of patients mTICI was greater than 2b without signs of secondary vessel injury. No vascular tissue was found in free clots of the aspiration samples.

**Conclusion:**

The examination of adherent tissue to the stentriever instead of the examination of free clots may affect the number of detected parts of vessel wall. Further studies in combination with vessel wall imaging may elucidate the origin of remnants of vessel wall.

## Introduction

Despite widespread use of retrieval devices dedicated to the treatment of large vessel occlusion (LVO) in acute ischemic stroke, little is known about the actual interaction of the stentriever and the vessel wall during the procedure. So far, several studies have reported on thrombus composition, but research has been primarily focused on the blood-derived components of the clots.^1–3^ Reports of atypical clot components such as atheroma or vascular endothelium suggestive of vessel wall injury are rare.^4,5^ However, trauma to the vessel endothelium has been formerly described in an animal model of mechanical thrombectomy (MT).^6,7^ In human, vascular wall components were scarcely reported in direct aspiration first pass technique (ADAPT) as well within combination with stentriever.^8^ Little is known about the direct interaction of the surface of the stentriever to the vessel wall.

The aim of this retrospective study was to investigate the involvement of the stentriever in vessel wall injury by analysis of the composition of adherent tissue to the stentriever (ATS) in absence of angiographic lesions during combined aspiration thrombectomy with stentriever (CATS).

## Material and methods

### Patients

Tissue material from CATS was retrospectively analyzed from patients who suffered an ischemic stroke. This study has been approved by the Ethic Committee of our institution. Written informed consent was obtained from all participants or their caregivers regarding the use of clinical, interventional, histological and imaging data for research purposes. Following clinical data were documented: gender, age cardiovascular risk factors, preexisting anticoagulation with or without atrial fibrillation, base line NHSS as well as preinterventional bridging lysis and the site of the occlusion. Stroke etiology was classified according to the TOAST criteria.^9^

### Interventional technique

All patients with stroke symptoms related to the site of the vessel occlusion and eligibility for MT were included. LVO was verified via CT angiography (CTA). Exclusion criteria were signs of ischemia in the complete vascular territory of the main trunk of the occluded vessel according to the ASPECT score.^10^ MT was performed by CATS either in conscious sedation or in general anesthesia. For the procedure, an aspiration catheter with or without a microcatheter with microwire was navigated to the occluded artery through a guiding catheter with continuous saline flush. Then a microcatheter and microwire was navigated to the distal site of the occlusion. Intravascular catheter position aside the thrombus was verified by contrast media injection. The stentriever was unfolded inside the thrombus. After a minimum of 3 min deployment, aspiration was initiated applying vacuum with a connected syringe to the aspiration catheter. The choice of the stentriever used was at the discretion of the interventional neuroradiologist. Four different types of stentriever were used in this study (Supplementary Table 1). Under continuous vacuum, the system of stentriever and aspiration catheter was pulled back until free flow of blood into the connected syringe was observed. If no free flow of blood through the aspiration catheter occurred, the guiding catheter was removed under continuous vacuum also.

### Histologic material

Clots from aspirates were collected routinely from all patients treated with MT. Randomly, stentriever with ATS was collected at the end of the thrombectomy. In patients with collected stentriever two different types of samples were distinguished: samples containing the tissue of the aspirates and a separate sample with the stentriever itself.

### Free clots

The syringe containing the aspirate was flushed gently through a pad and isolable tissue was identified as free clots and transferred to a formalin-filled container (4% buffered formaldehyde). If the removal of the aspiration catheter and/or the guiding catheter was necessary, the catheter was flushed and tissue was isolated in the same manner.

### Tissue adherent to the stentriever (ATS)

**Figure 1.**
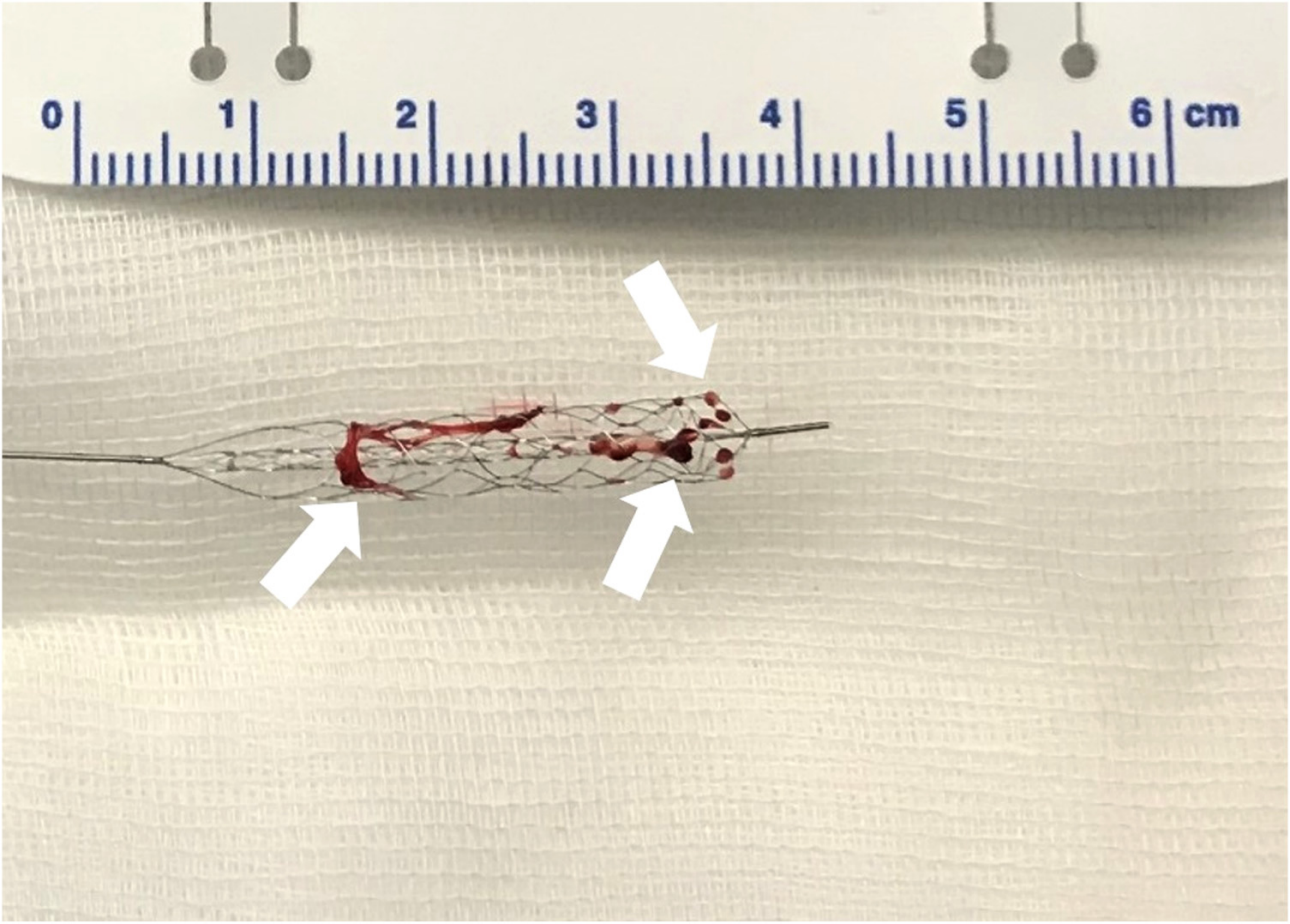
Tissue adherent to the stentriever (white arrows) used for histomorphological assessment.

After the final thrombectomy, the stentriever was dissected from the push wire and sampled separately for examination of ATS in a formalin-filled container (4% buffered formaldehyde) (Figure 1).

### Interventional data

The number of thrombectomy procedures and deployment time were analyzed and final angiographic results were classified using the Modified Treatment in Cerebral Ischemia (mTICI) score.^11^ The time of deployment is defined as the time in which the stentriever was inside unfolded in the thrombus. The onset time to last manoeuvre (OtlM) was the time from onset of stroke symptoms to the time point of the last retrieving manoeuvre.

### Histopathological analysis

**Figure 2.**
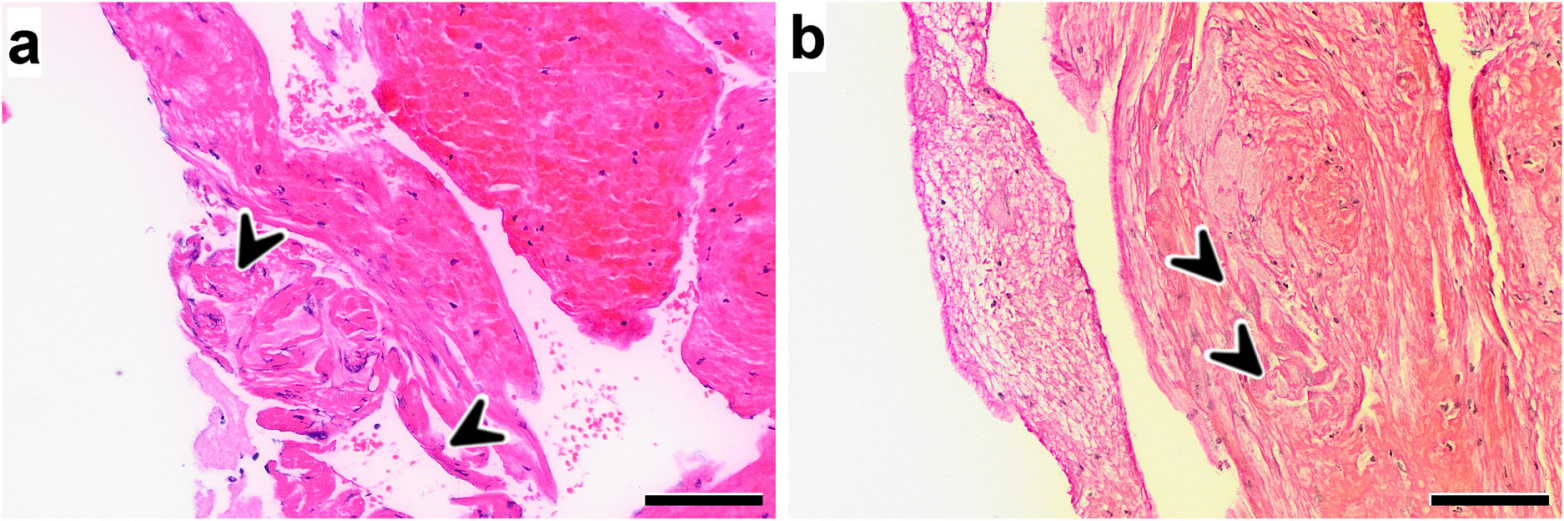
Stainings of a thrombus shows connective tissue as parts of vessel wall (arrowheads). Collagen bundles can be depicted in the EvG staining. Magnification 100×. Scale bar 100 μm. (a) Hematoxylin–Eosin stain (HE). (b) Elastica van Gieson stain (EvG).

**Figure 3.**
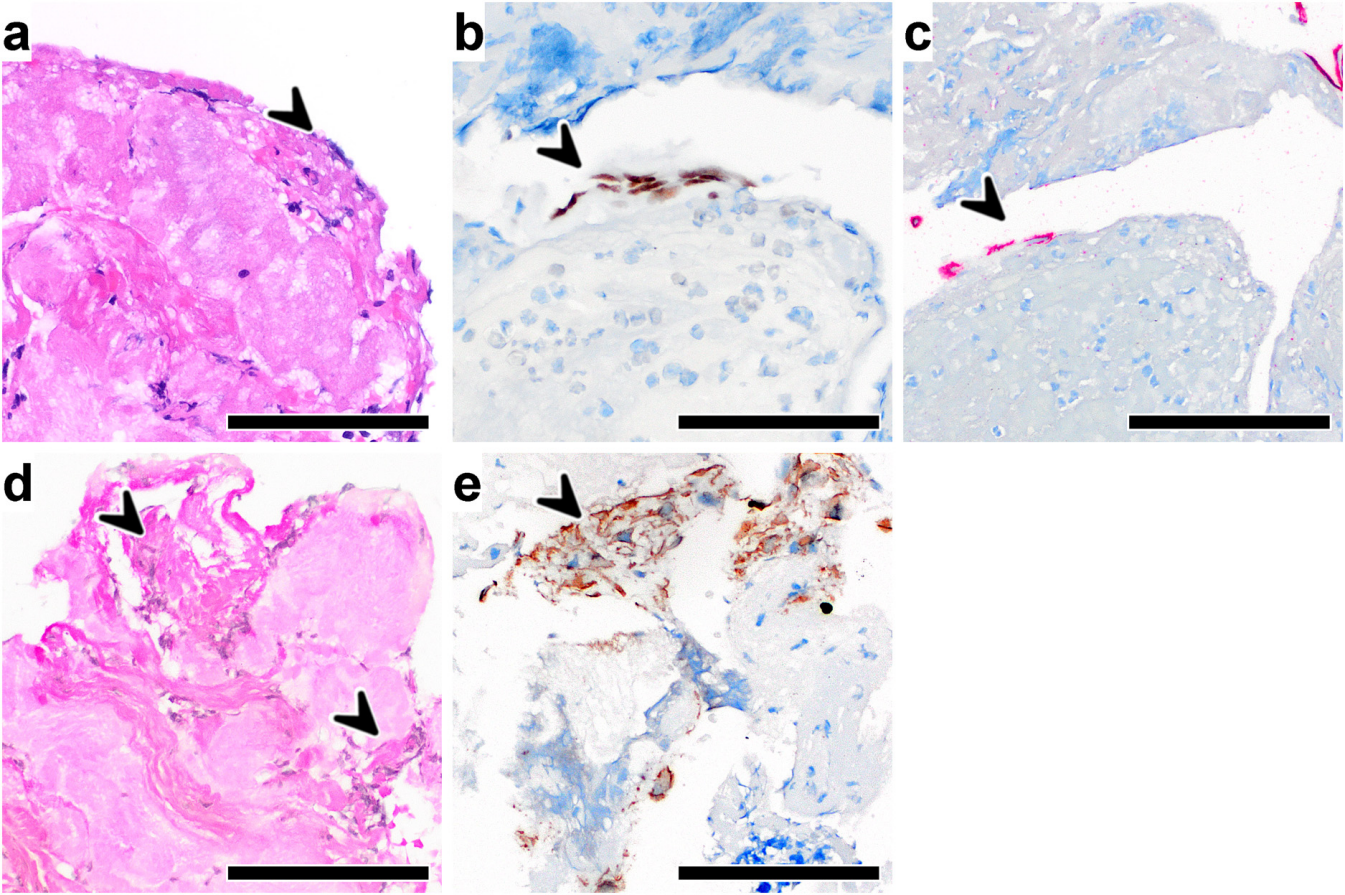
Immunohistochemical characterization of parts of the vessel wall (PVW) in thrombi obtained from stentriever. Magnification 200×, scale bar 100 μm. (a) HE staining of a thrombus containing PVW with flattened nuclei indicative of endothelial cell nuclei (arrowhead). (b, c) Immunohistochemical staining for ERG (a) and CD 34 and (c) highlighting the presence of endothelial cells (arrowhead) in the same thrombi imaged in panel a. (d) EvG staining of a thrombus containing parts of vessel wall visible by prominent red collagen tissue and sparse elastin fibers (arrowheads). (e) Immunohistochemical staining for SMA highlighting the presence of smooth-muscle cells (arrowhead) supposedly of the tunica media in the same thrombus imaged in Panel a–d.

Stentriever and free clots retrieved during MT were fixed in 4% buffered formaldehyde immediately after removal. Adherent tissue from the stentriever was removed carefully. Specimens from stentriever and aspirates were separately embedded in paraffin. 2 µm thick sections were cut followed by histological staining using Hematoxylin and Eosin (HE) and Elastica van Gieson (EvG) at the Institute of Pathology of our institution. Sections were categorized into an erythrocyte-rich thrombus (red), platelet-rich thrombus (white), or a mixed composition (Supplementary Figure 1) and scored with regard to organization and the presence of parts of vessel wall (PVW) by morphological assessment of HE and EvG stained sections. PVW were defined as either visible connective tissue in HE or EvG staining (Figure 2) or as immunohistochemical detection of CD34, SMA or ERG positive cells (Figure 3). Immunohistochemistry for smooth muscle actin, dilution 1:100 (M0851, Agilent Dako, California, USA), CD34, ready-to-use(RTU) (790-2927, Hoffmann-La Roche, Basel, Swiss) and ERG, dilution 1:20, (CM 421 C, Biocare Medical, California, USA) were stained on a VENTANA BenchMark ULTRA (Hoffmann-La Roche, Basel, Swiss) platform with the aforementioned dilutions, pretreatment and incubation times according to the manufacturer's advice. The histological analysis was performed blinded to clinical data and recanalization results on a routine diagnostic light microscope (BX43, Olympus, Tokyo, Japan). Representative images were acquired with an Olympus CS50 camera (Olympus, Tokyo, Japan) using Olympus cellSens Software (Olympus, Tokyo, Japan) on the above mentioned routine diagnostic light microscope. Image processing was carried out in ImageJ software.^12^

### Ethics approval

The patients or their caregivers gave written informed consent to the scientific use of the data. This study has been approved by the Ethic Committee of our institution.

## Results

### Patients

In 21 out of 238 patients with occlusion in the anterior and posterior cerebral circulation which were treated between April 2015 until November 2018 with SAT, a separate sample of the stentriever was available. From these 21 patients, two types of specimens were collected: free clots from the SAT manoeuvre(s) and samples with the stentriever itself after the final maneuver. Although the number of included patients was too small for a valid statistical analysis, regarding the proportion of patients with PVW + and PVW- specimens concerning age, cardiovascular risk factors, such as hypertension, diabetes and atrial fibrillation seems to be equal as it seems to medical treatment before stroke intervention (e.g., therapy with anticoagulants). Patients with PVW + specimens tended to have a higher pretherapeutic score in the National Institutes of Health Stroke Scale (NHISS). The patient characteristic, preinterventional treatment, cardiovascular risk factors, stroke etiology and the site of occlusion of the study population enrolled are shown in Table 1.

**Table 1.** Patients characteristics.

Patients with		Stent without PVW	Stent with PVW
		14	7
Male		9 (64.2)	5 (71–4)
Age on admission (years)		72.5 (49.7–91.2)	76.8 (58.24–94)
Hypertonus		9 (64.2)	5 (71.4)
Diabetes		4 (28.6)	2 (28.6)
Atrial fibrillation		8 (57.1)	5 (71.4)
Anticoagulation		6 (42.9)	4 (57.1)
Baseline NIHSS		14 (9–22)	16 (12–20)
**Stroke etiology (TOAST)**			
Cardioembolic/LAA/Undetermined		9/1/4	6/0/1
**Pre-interventional treatment**			
Bridging lyse		9 (64.2)	4 (57.1)
General anesthesia		11 (78.6)	6 (85.7)
**Location of occlusion**			
ACI		4 (28.6)	1 (14.2)
A2		1 (7.1)	0 (0)
M1		5 (35.7)	4 (57.1)
M2		3 (21.4)	2 (28.6)
Basilar artery		1 (7.1)	0 (0)

Values are n (%) or median (range). ICA = internal carotid artery; LAA = large artery atherosclerosis; PVW = parts of vessel wall; StTr = stentriever.

### Treatment and Outcome

The percentage of use or waiver of systemic intravenous recombinant tissue plasminogen activator (rTPA) (“Bridging Lysis”) prior to the thrombectomy procedures, and the use or waiver of general anesthesia for the thrombectomy procedure and the median number (n = 2) of thrombectomy maneuvers did not differ between both groups, with a range of 1–10 maneuvers in PVW- and a range of 1 to 4 maneuvers in PVW + patients. The outcome concerning mTICI score was better than mTICI 2b in more than 90% of patients in both groups without angiographic signs of secondary vessel injury e.g., dissection. Finally, no difference appeared in the onset time to last manoeuvre (OtlM) was found (Table 2).

**Table 2.** Interventional data and outcome.

Interventional n = 21	StTr without PVW	StTr with PVW
	n = 14	n = 7
**Manoeuvres: **	2(1–10)	2(1–4)
1	7 (50)	5 (71.4)
2	4 (28.7)	0 (0)
3	1 (7.1)	0 (0)
4	1 (7.1)	1 (14.3)
5	0 (0)	1 (14.3)
10	1 (7.)	0 (0)
**Stentriever**		
**Type **	**n (%)**	**n (%)**
1	1 (7.1)	0 (0)
2	11 (78.6)	6 (85.7)
3	0 (0)	1 (14.3)
4	2 (14.3)	0 (0)
**Time of Deployment: minutes**		
≤ 3	12 (85.7)	6 (85.7)
4	2 (14.3)	0 (0)
6	0 (0)	1 (14.3)
**mTICI** ** ≧2b: **	13 (92.8)	7(100)
2a	1 (7.1)	0 (0)
2b	6 (42.9)	4 (57.1)
3	7 (50)	3 (42.9)
**Time of Procedure: OtlM (h:min)**	5:08 (2:03–7:13)	4:10 (2:29–6:05)

Values are n (%) or median, Deployment time is in minutes (min). Manoeuvres: Number (n) of combined aspiration thrombectomy with stentriever. Stentriever (Type) = see Supplement S1. Deployment time = Time in which the stentriever was inside unfolded in the thrombus. OtlM = Onset time to last Manoeuvre. StTr = Stentriever (Type see Supplement Table S1).

### Histopathological results

#### Free clots

The aspirates containing free clots of all 21 patients consisted of fresh thrombus components without signs of thrombus organization (Table 3).

**Table 3.** Histopathologic characteristic of tissue from stentriever.

Histopathologic characteristic of tissue from stentriever n = 7							
**ID**	**1**	**2**	**3**	**4**	**5**	**6**	**7**
PVW location	StTr	StTr	u/o	StTr	StTr	StTr	u/o
White thrombus	**+**	**−**	**−**	**−**	**−**	**−**	**−**
Red thrombus	**−**	**−**	**−**	**−**	**−**	**−**	**+**
Mixed thrombus	**−**	**+**	**+**	**+**	**+**	**+**	**−**
Atheroma	**−**	**−**	**−**	**−**	**−**	**−**	**−**
Connective tissue (EvG)	**+**	**+**	**+**	**+**	**+**	**+**	**+**
**Thrombus age**							
Fresh	**+**	**+**	**+**	**+**	**+**	**+**	**+**
Organized	**−**	**−**	**−**	**−**	**−**	**−**	**−**
**Immunohistochemistry**							
SMA	**−**	**+**	**−**	**−**	**−**	**−**	**+**
CD34	**−**	**+**	**+**	**−**	**−**	**+**	**−**
ERG	**−**	**+**	**−**	**−**	**−**	**−**	**+**

CD34 = CD34 antibody; Connective tissue (EvG) = Collagen and/or elastic fibers proved by EvG stain; ERG = Transcriptional regulator ERG (V-ets erythroblastosis virus E26 oncogene homolog); SMA = Smooth muscle actin; StTr = Stentriever; u/o = Unknown origin due to technical issues during histological examination.

#### Adherent tissue to the stentriever (ATS)

In 7 out of 21 patients, the samples contained PVW identified by presence of connective tissue in HE stain and/or collagen in EvG stain (Figure 2). Further analysis of these 7 specimens via immunohistochemistry revealed the presence of both endothelial cells (CD34+ and/or ERG+) in 4 out of 7 cases (ID 2, 3, 6 and 7) and smooth muscle cells of the vascular wall (Sm-Actin+) in 2 out of 7 cases (ID 2 and 7). In three of the seven cases the diagnosis of vessel wall remnants was made by conventional staining (EvG) only (ID 1, 4 and 5) (Figure 3). In 2 out of 7 cases, loose PVW were found in the specimen container (ID 2 and 7), in which due to technical issues during sample preparation, the exact localization (stentriever adherent vs. aspirate located) of the PVW could not be determined. The aspirates of the remaining 19 patients were negative for remnants of vessel wall and/or atheromatous debris by morphological assessment of HE and EvG stained sections (Table 3).

## Discussion

The aim of this retrospective study was to investigate the adhesion of vascular tissue to the stentriever from patients who received combined MT. In summary, we found remnants of vessel wall in 7 out of 21 (33%) patients ATS. All free clots were free of remnants of vessel wall.

**Figure 4.**
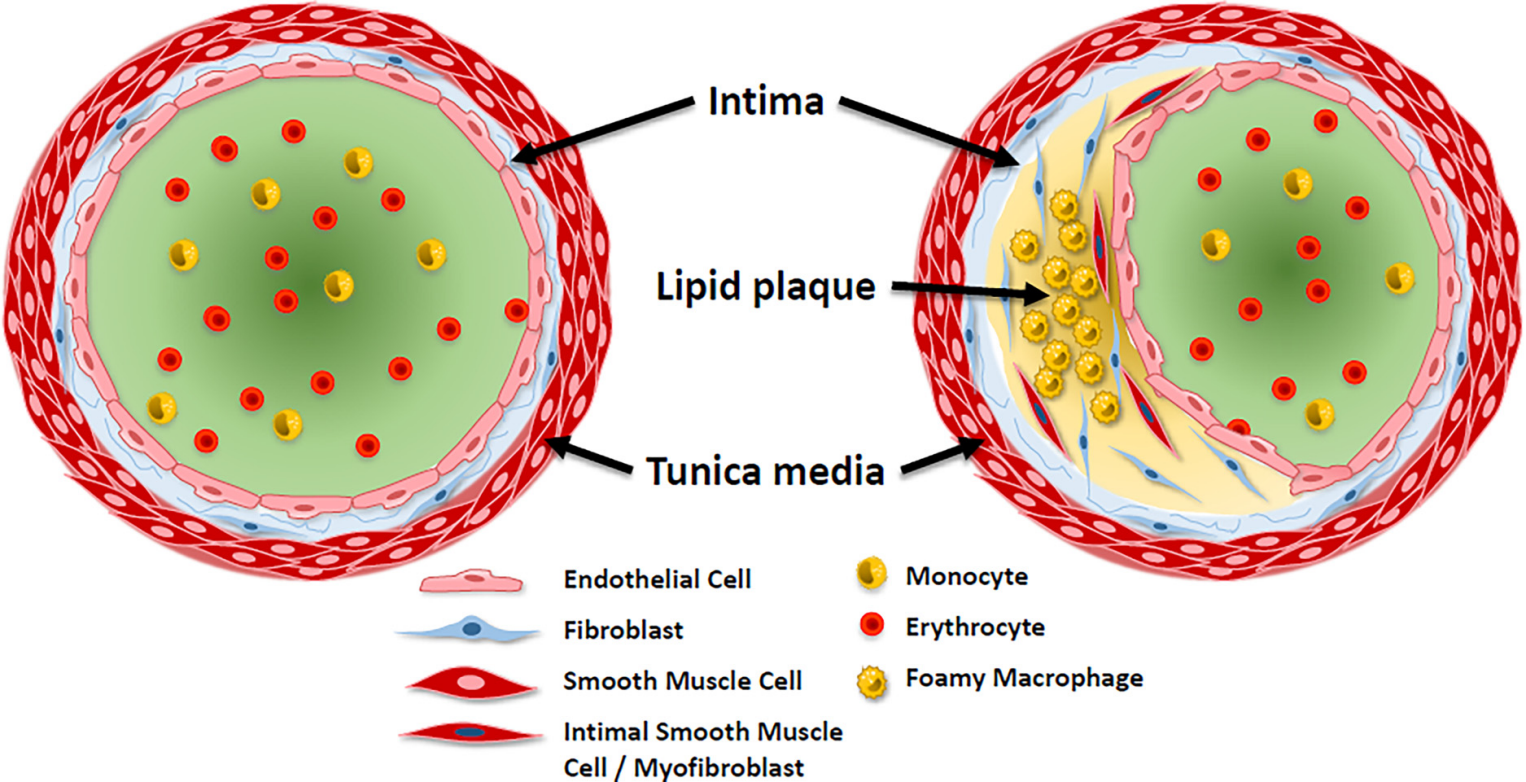
Schematic drawing of histopathologic changes in arteriosclerotic blood vessels. In a regular healthy artery (left) smooth muscle cells, that express smooth-muscle actin (SMA), only exist in the tunica media. The inner layer of the blood vessel, the intima, is thin and consists of scarce fibroblast and collagen matrix. In an arteriosclerotic artery (right), however, the intima is thickened, partially obliterating the lumen and filled with lipid laden foamy macrophages, fibroblasts and several smooth muscle cells and myofibroblasts, both expressing SMA, the so-called arteriosclerotic plaque. These compounds can end up in the stentriever (due to damage of the Intima) or the thrombus itself (due to plaque rupture). Therefore, the presence of SMA-expressing cells in the herein presented samples is not indicative of deeper blood-vessel damage of the Tunica media.

In 2006, Marder reported a small atheroma with attached arterial intima and subintima from a human cerebral artery in a specimen derived from thrombectomy, using the Merci System.^4^ In different animal models testing various neurovascular MT devices, vessel wall injury has been a frequent finding. The in vivo removal of artificial clots by with wall-contact devices with or without aspiration in swine^6,13^ or the ex-vivo examination of rabbit carotid artery.^14^ In human, Fonatsu found components of vessel wall in 24 out of 150 clot specimens according to 22 out of 101 patients with ischemic stroke.^8^ In a recent study, atypical thrombus content with PVW was detected in 4 out of 302 patients and atheroma in another 4 out of 302 patients in thrombectomy aspirates.^15^ As proposed by Singh in 2013, the detection of subendothelial connective tissue in thrombi retrieved by MT is consistent with subendothelial injury of cerebral arteries.^5^ Additionally, studies in post-interventional MRI imaging also reported indirect signs of vessel wall injury, with higher rates of injury in patients treated with a stentriever system compared to contact aspiration.^16^ In 7 out of 21 patients included in our study, PVW were identified by the presence of connective tissue in the form of collagen and/or elastic fibers in Elastic-van Gieson (EvG) in the adherent tissue at the stentriever. In immunohistochemical staining, specimens in 4 patients were positive for CD34 and/or ERG which were considered as markers for vascular endothelium. Two specimens were positive for smooth muscle actin (SMA+). In a healthy artery, SMA+ smooth muscle cells are only found in the tunica media. In arteriosclerotic vessels, however, during plaque growth, smooth muscle cells migrate into the arteriosclerotic lesion increasing the intraplaque collagen content, especially in the so-called fibrous cap (Figure 4).^17^ If plaques become unstable, the number of smooth muscle cells and collagen content decreases in the fibrous cap, leading to a vulnerable state increasing the risk of rupture.^18,19^ In the media of arteriosclerotic arteries, a severe reduction of smooth muscle cells, together with an increased collagen content can be found.^20^ Thus, the detection of SMA + cells in the adherent parts can be the result of an injury of an altered intima, the arterýs tunica media, or the destruction of the fibrous cap of an arteriosclerotic plaque. In accordance with the findings of Mereuta all aspirate specimens, with or without PVW, were fresh thrombi without findings of atheromatous tissue or signs of thrombus organization indicating, that the adherent PVW at the stentriever are due to the shearing forces of the stentriever itself.^21^ Renu discussed a damaging effect of bridging lysis in addition to the stress to the vessel wall by the retrieving devices.^22^ This “synergistic” injury cannot be excluded in our study, since the proportion of patients with and without pre-interventional bridging lysis was equal in both groups. All types of stentriever used in this study consisted of the uncoated titanium alloy nitinol with known thrombogenic potential.^23^ No stentrievers were surface-treated to prevent clot formation. Accordingly, thrombogenicity of the stentriever may explain adherent clot-formation on the struts of the stentriever. On the other hand, thrombus-formation on nitinol is a time dependent process^24^ and does not explain the reported PVW. Patients with PVW + specimens tended to have a higher pretherapeutic score in the National Institutes of Health Stroke Scale (NHISS). This trend might hint at a relevance of ischemia, not only to the brain tissue, but also to the occluded arteries and would further explain the higher rate of PWV in patients with high NHISS due to ischemic intimal and subintimal damage. The examination of adherent tissue to the stentriever resulted in a high amount of PVW in our study. Lower findings of remnants of vessel wall in the literature may be explained by limitation of the histopathological examination to free clots in former studies^7,18^ in contrary to additional examination of adherent tissue to the stentriever in our study. Finally, we postulate that the surface properties of the stentriever cause adhesion of the clot to the stentriever, a desired capability for thrombus removal. Together with shearing forces to the altered vessel wall, the surface properties of the stentriever might explain the adherence of PVW to the stentriever.

### Limitations

The main limitation of our study is its small sample size. A larger sample would provide more robust evidence about the influence of the technique and devices used in MT. The histomorphological examination of the stentriever was performed after the final thrombectomy procedure. Therefore, we cannot exclude that in foregoing manoeuvres PVW went upstream in more peripheral arterial segments, and could thus not be detected by histologic analysis. By flushing the aspirate through the pad for the histological examination it may be possible that invisible PVW remain in the pad. This would lead to false negative detection of PVW in the aspirates. In addition, the preparation of adherent tissue from the stentriever struts is a delicate process and besides PWV other readouts (e.g., thrombus composition) could have an influence on procedure and patient outcome.^25^ All types of stentriever used had different strut designs and variable dimensions, but all contained uncoated nitinol. So, we cannot rule out clot-formation due to the stentriever surface itself. Finally, the presence of SMA-expressing cells in the herein presented samples are not indicative of deeper blood-vessel damage of the Tunica media due to possible intimal SMA+ positive cells in arteriosclerotic arteries.

## Conclusion

The examination of adherent tissue to the stentriever instead of the examination of free clots may affect the number of detected PVW. The high number of remnants of vessel wall ATS in this limited study might be due to the surface properties of the stentriever. Consequently, this may explain the high findings of vessel wall injury in our study in comparison to the examination of free clots only after MT reported in the literature. This finding underlines the need of further improvement of stentriever design for tissue-conserving thrombus removal thus preventing secondary damages e.g., subarachnoid hemorrhage or dissection of the target vessel.

## Supplemental Material

sj-tif-1-ine-10.1177_15910199231216764 - Supplemental material for Adhesion of vessel wall to stentriever during combined technique for mechanical thrombectomy in acute ischemic stroke: A histomorphological study Supplemental material, sj-tif-1-ine-10.1177_15910199231216764 for Adhesion of vessel wall to stentriever during combined technique for mechanical thrombectomy in acute ischemic stroke: A histomorphological study by Frank Donnerstag, Christopher Werlein, Friedrich Götz, Omar Abu Fares, Peter Raab, Enrico Calvino Iglesias, Heinrich Lanfermann, Mike P Wattjes and Danny Jonigk in Interventional Neuroradiology

sj-docx-2-ine-10.1177_15910199231216764 - Supplemental material for Adhesion of vessel wall to stentriever during combined technique for mechanical thrombectomy in acute ischemic stroke: A histomorphological study Supplemental material, sj-docx-2-ine-10.1177_15910199231216764 for Adhesion of vessel wall to stentriever during combined technique for mechanical thrombectomy in acute ischemic stroke: A histomorphological study by Frank Donnerstag, Christopher Werlein, Friedrich Götz, Omar Abu Fares, Peter Raab, Enrico Calvino Iglesias, Heinrich Lanfermann, Mike P Wattjes and Danny Jonigk in Interventional Neuroradiology
