# Supplementary figures and images for: Adhesion of vessel wall to stentriever during combined technique for mechanical thrombectomy in acute ischemic stroke: A histomorphological study
Source: Interv Neuroradiol. 2023 Dec 6;32(3):472–9. doi: 10.1177/15910199231216764 (PMC13294579; doi:10.1177/15910199231216764)

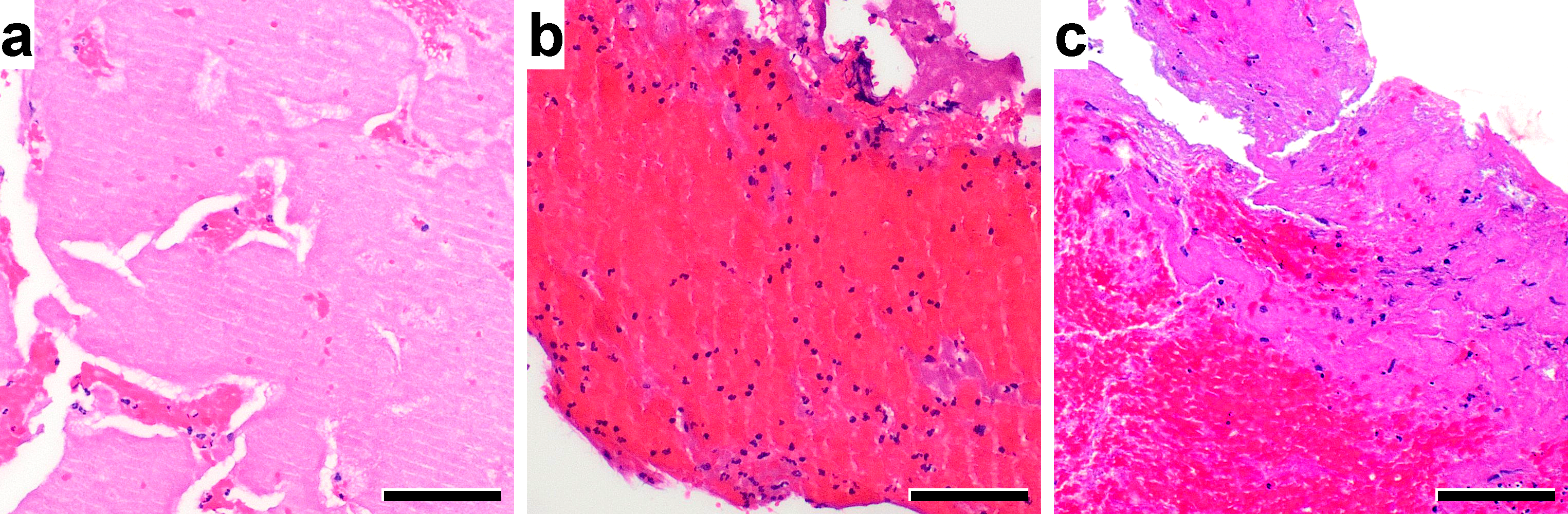

Supplement: sj-tif-1-ine-10.1177_15910199231216764 - Supplemental material for Adhesion of vessel wall to stentriever during combined technique for mechanical thrombectomy in acute ischemic stroke: A histomorphological study [file sj-tif-1-ine-10.1177_15910199231216764.tif]
