# Supplementary material for: Adhesion of vessel wall to stentriever during combined technique for mechanical thrombectomy in acute ischemic stroke: A histomorphological study
Source: Interv Neuroradiol. 2023 Dec 6;32(3):472–9. doi: 10.1177/15910199231216764 (PMC13294579; doi:10.1177/15910199231216764)
Supplement: sj-docx-2-ine-10.1177_15910199231216764 - Supplemental material for Adhesion of vessel wall to stentriever during combined technique for mechanical thrombectomy in acute ischemic stroke: A histomorphological study [file sj-docx-2-ine-10.1177_15910199231216764.docx]

**Supplement 1: Used Stent Retriever:**

| **ID Stentriever** | **Commercial Name and Producer of Stentriever** |
| --- | --- |
| 1 | Embotrap II 5mm/20mm: Cerenovus/Neuravi, Galway, Ireland |
| 2 | Solitaire: FR 4mm/20mm (Micro Therapeutics Inc. /ev3 Neurovascular, Irvine, California USA |
| 3 | Aperio 4.5mm/30mm: Acandis GmbH Pforzheim, Germany |
| 4 | pREset 4mm/20mm: phenox GmbH, Bochum, Germany |
